# “Partial Open Sky Method” - A novel technique to avoid the open sky condition during Triple procedure or Penetrating keratoplasty

**DOI:** 10.22336/rjo.2023.61

**Published:** 2023

**Authors:** Jaya Kaushik, Sunandan Bhatta, Ankita Singh, Rakesh Jha

**Affiliations:** * Department of Ophthalmology, Command Hospital (Lucknow), U.P, India; ** Department of Ophthalmology, Military Hospital (Agra), U.P, India; *** Department of Ophthalmology, Military Hospital (Bathinda), Punjab, India

**Keywords:** penetrating keratoplasty, open sky procedure, suprachoroidal hemorrhage

## Abstract

Penetrating keratoplasty is referred to as an “open-sky” procedure because the intraocular contents are entirely exposed to atmospheric pressure after the diseased cornea has been trephined off and before the donor button is sutured. Suprachoroidal hemorrhage (SCH) is a major vision-threatening complication, associated with this open-sky procedure. While numerous factors may predispose an eye to SCH, like hypertension, myopia, trauma, glaucoma, etc., it is better to be prepared for the worst eventuality.

We described a novel technical modification, denoted as the “partial open sky technique”, that we used during the surgical steps of trephining and excision of host corneal tissue in seven cases of triple procedure and penetrating keratoplasty in our center over two months. We propose that the technique would be additionally helpful in managing the inadvertent suprachoroidal hemorrhage associated with keratoplasty over the available existing methods.

**Abbreviations:** SCH = Suprachoroidal hemorrhage, ICCE = Intracapsular cataract surgery, ECCE = Extracapsular cataract surgery, WTW = White to White

## Introduction

Penetrating keratoplasty is referred to as an “open-sky” procedure because the intraocular contents are entirely exposed to atmospheric pressure after the diseased cornea has been trephined off and before the donor button is sutured. Suprachoroidal hemorrhage (SCH) is a major vision-threatening complication, associated with this open-sky procedure [**1**]. Although rare, this complication is devastating to the patient as well as to the operating surgeon because of the expertise required to manage it rapidly by corneal surgeons.

With procedures like ICCE and large incision ECCE going almost to near oblivion, penetrating keratoplasty is the only remaining elective open-sky procedure in today’s era, if open-globe injuries are set aside [**2**]. Hence, prophylaxis and safeguarding against this dreaded SCH are of utmost importance.

While numerous factors like hypertension, myopia, trauma, glaucoma, etc. may predispose an eye to SCH, it is better to be prepared for the worst eventuality [**3**,**4**]. 

We described a novel technical modification, called the “partial open sky technique”, that we used during the surgical steps of trephining and excision of host corneal tissue in seven cases of triple procedure and penetrating keratoplasty in our center over two months. We propose that the technique would be additionally helpful in managing the inadvertent suprachoroidal hemorrhage associated with keratoplasty over the available existing methods.

## Surgical Technique

While there have been reports of methods for the avoidance of the open sky situation during penetrating keratoplasty, they are either time-consuming or technically challenging. There is barely any learning curve for novice cornea surgeons to do the treatment as it is now described, which is a small modification of an established technique. A similar technique has not been described before.

The host cornea is trephined using a handheld trephine, the size of which is determined by the usual criteria of White to White (WTW) and size of opacity. Using the trephine, a partial thickness cut is made preferably up to the pre-Descemet’s region. A full-thickness entry is made at the 11 o’clock position using a 15o side port. Exit of aqueous is observed to ensure actual entry into the anterior chamber. The AC is then filled with OVD and the incision is enlarged both on right and left sides using right and left corneal scissors. A one-clock-hour area of the recipient cornea between 3 to 4 o’clock is left uncut.

**Fig. 1:**
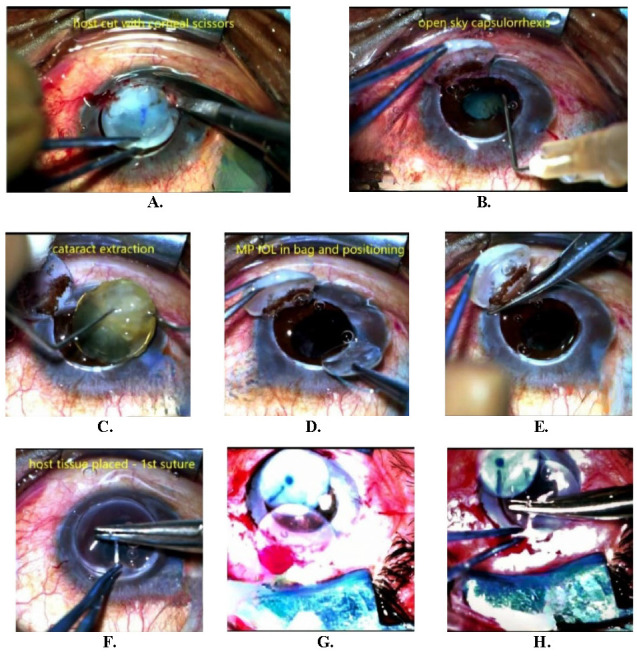
Clinical images of the surgical steps during the Partial Open Sky technique (**A-H**). After trephining the host, dissection is continued in the usual way leaving one clock hour of attachment (**A**). Cataract extraction is then carried out in partial open sky after reflecting the flap-capsulorhexis (**B**), lens extraction (**C**), and 3-piece rigid IOL implantation (**D**). The flap is thereafter cut, and the graft is placed and proceeded with the usual sequence (**E-F**). In case the vitreous push is encountered, the diseased host is removed only after the graft has been slid into position and the first cardinal suture placed (**G-H**)

The diseased tissue is then held with Lim’s forceps at 9 o’clock and turned out nasally for the right side or temporally for the left-sided case; with the uncut region acting as a “hinge - the partial open sky”. The comforting aspect of this technique is the reassurance of being able to close the “sky” immediately at the slightest hint of vitreous prolapse (**Fig. 1 C**).

In the case of triple procedure, capsulorhexis, hydro procedures, lens extraction, irrigation-aspiration, and placement of PCIOL are done with the partial open sky, the tissue either held by the surgeon with the left hand or by the assistant.

In case no complications are met and none further are anticipated, the diseased host tissue is dissected out completely before putting in the optical donor graft. However, in high-risk cases like noninfectious conditions needing tectonic PK, if the vitreous push is felt, the donor graft is slid under the cover of the host cornea. The first cardinal suture is placed at 12 o’clock. The diseased host is then removed, and the rest of the penetrating keratoplasty is carried out as usual.

While there have been reports of methods for the avoidance of the open sky situation during penetrating keratoplasty, they are either expensive, time-consuming, or technically challenging. There is barely any learning curve for a cornea surgeon to do the treatment as it is now described, which is a small modification of an established technique. A similar technique has not been described before.

## Results

The technique was introduced as a novel step during the triple procedure/Optical penetrating keratoplasty/noninfectious conditions requiring tectonic PK, done by a single surgeon between Jan 2023 and Feb 2023. A total of 7 cases were operated, 5 of which were triple procedures and the remaining 2 were penetrating keratoplasties. 

No complications of vitreous push or expulsive choroidal hemorrhage were encountered in any case. In one case, when an apparent vitreous push was felt after cataract extraction under partial open sky, the surgeon was able to quickly place the donor graft under the cover of the host tissue and put the first cardinal suture before dissecting out the host tissue.

There was no appreciable increase in surgery time due to the incorporation of the step. Feedback from the operating surgeon was positive in terms of reduced surgeon anxiety level during the procedure and increased control during cataract extraction. Some technical expertise and familiarity with the technique were required when and if placing the donor graft under the cover of the host tissue and placement of the first cardinal suture. No unique attributable complications were encountered in the cases in which the technique was incorporated. 

## Discussion

Supra-choroidal hemorrhage (SCH) is a complication dreaded by all ophthalmic surgeons as it strikes at a time least expected and any delay in treatment might result in an eye that is blind or necessitates enucleation or evisceration. Variously named Suprachoroidal hemorrhage (SCH), Suprachoroidal hematoma, Acute intraoperative suprachoroidal hemorrhage, Expulsive choroidal hemorrhage, etc. [**1**], the condition is defined as the accumulation of blood between the choroid and the sclera [**2**]. If not promptly addressed, it can result in rapid expulsion of all intraocular contents culminating in loss of vision or even loss of eye - the so-called massive suprachoroidal hemorrhage [**3**]. Its incidence during penetrating keratoplasty has been variously reported to range between 0.45% to as high as 2% [**4**-**7**]. 

Presently, the technique available for the intraoperative management of SCH is wound closure, deepening of the anterior chamber using a viscoelastic agent/heavy liquids, and creation of a surgical drainage by posterior sclerotomy. In all these procedures, the key to limiting the progress of the ECH is the rapidity with which wound closure is created and the tamponade effect is obtained. The “hinged flap” in our technique would be “readily available” to the surgeon and wound closure may be obtained earlier with simple re-suturing of this hinged flap to the recipient’s excised corneoscleral bed. Suturing of this hinged flap would be better opposed and would create a better tamponade effect compared to the conventional use of donor corneal tissue, as it is usually oversized and may be left with subtle wound leak due to rapid suturing as required in case of SCH and a consequent cycle of persistence of hypotony and aggravation of SCH. 

Secondly, the donor corneal tissue may be removed from the bed (if sutured partially at the onset of SCH), and this precious tissue may be reserved for performing keratoplasty in other patients in need.

Thirdly, the wound closure obtained with the recipient’s cornea in such cases would impose reduced post-operative inflammation compared to when using the donor corneal tissue, as the donor tissue in the early stage is usually edematous and might incite post-operative inflammation. The opposition may not be of adequate strength because of post-operative inflammation, especially in cases of acute graft rejection. 

Additionally, the technique requires a negligible learning curve, with no requirement for any specialized instruments or additional surgical tissue.

Lastly, the availability of tissue instantaneously for any untoward events may be instrumental in reducing the anxiety level among the novice corneal surgeon, while performing the keratoplasty, especially during the entire stage of “open-sky” created after recipient corneal button excision.

Despite, the several advantages, our technique is limited by the factor that it may not be useful in cases of therapeutic keratoplasty because of the risk of contamination of the donor graft. 

Besides, the technique was applied on a limited number of patients, with no significant effect on reducing hypotony and we did not encounter any SCH while performing keratoplasty using this technique, the real-time experience and follow-up data with this technique aimed for is not available to share. Because of limited experience, we propose that a larger series or RCT with standard technique as comparison or multi-centric study in this technique would be crucial to substantiate our results.

## Conclusion

The technique of partial open sky by the creation of a temporary hinged flap of recipient corneal tissue during penetrating keratoplasty may be helpful to instantly cover the surgical defect in case of inadvertent suprachoroidal hemorrhage. The technique is simple to perform with no requirement for specialized instruments or additional surgical tissue.

**Conflict of Interest Statement**

The authors state no conflict of interest. 

**Informed Consent and Human and Animal Rights Statement**

Informed consent has been obtained from the patients included in this study.

**Authorization for the use of human subjects**

Ethical approval: The research related to human use complies with all the relevant national regulations and institutional policies, as per the tenets of the Helsinki Declaration, and has been approved by the Ethics Committee of Military Hospital (Bathinda), Punjab, India.

**Acknowledgments**

Hav SK Singh, Bachelor in Paramedical Training (Optometry) for technical help.

**Sources of Funding**

None.

**Disclosures**

None.
